# Immunoglobulin A (IgA)-Mediated Vasculitis as a Paraneoplastic Clue in Chronic Lymphocytic Leukemia: A Case Report

**DOI:** 10.7759/cureus.110005

**Published:** 2026-05-31

**Authors:** Sai Sushrutha Mudupula Vemula, Soumith Sanka, Dheeraj Peddinti, Ujwala Koduru, Richa Tikaria

**Affiliations:** 1 Internal Medicine, Sparrow Hospital, Lansing, USA; 2 Internal Medicine, Michigan State University College of Osteopathic Medicine, East Lansing, USA; 3 Internal Medicine, Kansas City University, Kansas City, USA; 4 Hematology/Oncology, Michigan State University College of Human Medicine, East Lansing, USA; 5 Internal Medicine, Michigan State University, East Lansing, USA

**Keywords:** chronic lymphocytic leukemia, cll, iga vasculitis, immune system dysregulation, para neoplastic phenomenon

## Abstract

Immunoglobulin A (IgA)-mediated vasculitis is a small-vessel vasculitis defined by IgA-dominant immune complex deposition. Although common in children, adult-onset cases are rare and may signal underlying malignancies. In this case report, we present a 60-year-old male with chronic lymphocytic leukemia (CLL) under observation for his painful purpura and non-healing leg ulcers, with a biopsy confirming IgA vasculitis. His course was further complicated by steroid-induced diabetes, poor wound healing, recurrent infections, and fatal respiratory failure. He remained steroid-dependent with recurrent ulcer flares on tapering and infections, including pneumonia and varicella-zoster. Flow cytometry revealed persistent clonal B-cell populations with stable lymphocyte counts, suggesting subclinical CLL-driven immune dysregulation. This case highlights adult-onset IgA vasculitis as a potential paraneoplastic clue of indolent CLL even in the absence of leukemia progression. It underscores the importance of early malignancy screening, coordinated multidisciplinary care, and early steroid-sparing therapy in adult vasculitis patients with atypical features.

## Introduction

Immunoglobulin A (IgA) vasculitis (formerly Henoch-Schönlein purpura (HSP)) is a small-vessel vasculitis classically presenting with palpable purpura, arthralgia, renal involvement, and abdominal symptoms, and leukocytoclastic vasculitis with IgA-dominant immune deposits on histology [1]. IgA vasculitis occurs predominantly in children (incidence 3-26.7/100,000), with a significantly lower adult incidence of 0.8-1.8/100,000 [2], and should prompt an evaluation for an underlying systemic disease, including autoimmune disorders, solid neoplasia, and hematologic malignancy [3]. IgA vasculitis shows a fall-winter predominance in children and a bimodal summer-winter pattern in adults, occurs worldwide across all ethnic groups, but has a significantly lower annual incidence in Black children compared with White or Asian children [4,5].

Chronic lymphocytic leukemia (CLL) is the most common adult leukemia in Western countries, frequently diagnosed at around 65-70 years of age, and around 50% of patients remain asymptomatic without requiring any treatment [6,7]. Currently, the most widely used prognostic markers are heavy chain variable region mutations (IGHV), zeta-associated protein-70 (ZAP-70), expression of CD38, and indolent clonal proliferation of mature CD5+ CD19 B lymphocytes [6,7]. The treatment of CLL involves the introduction of novel therapeutic agents, combination chemotherapy, allogeneic stem cell transplant, and precision medicine. It is frequently associated with immune dysregulation, including autoimmune hemolytic anemia (~7%) and immune thrombocytopenia (<1-2%) [8]. Beyond these cytopenias, CLL may also be associated with other rare systemic autoimmune syndromes, including IgA leukocytoclastic vasculitis [8,9].

The overlap between autoimmune vasculitis and CLL represents an understudied, but clinically significant, intersection between lymphoproliferative and autoimmune diseases. Adult-onset IgA or antineutrophil cytoplasmic antibody (ANCA) vasculitis may serve as a paraneoplastic warning for prompt evaluation and consideration of an underlying hematologic malignancy like CLL [9].

## Case presentation

**Figure 1:**
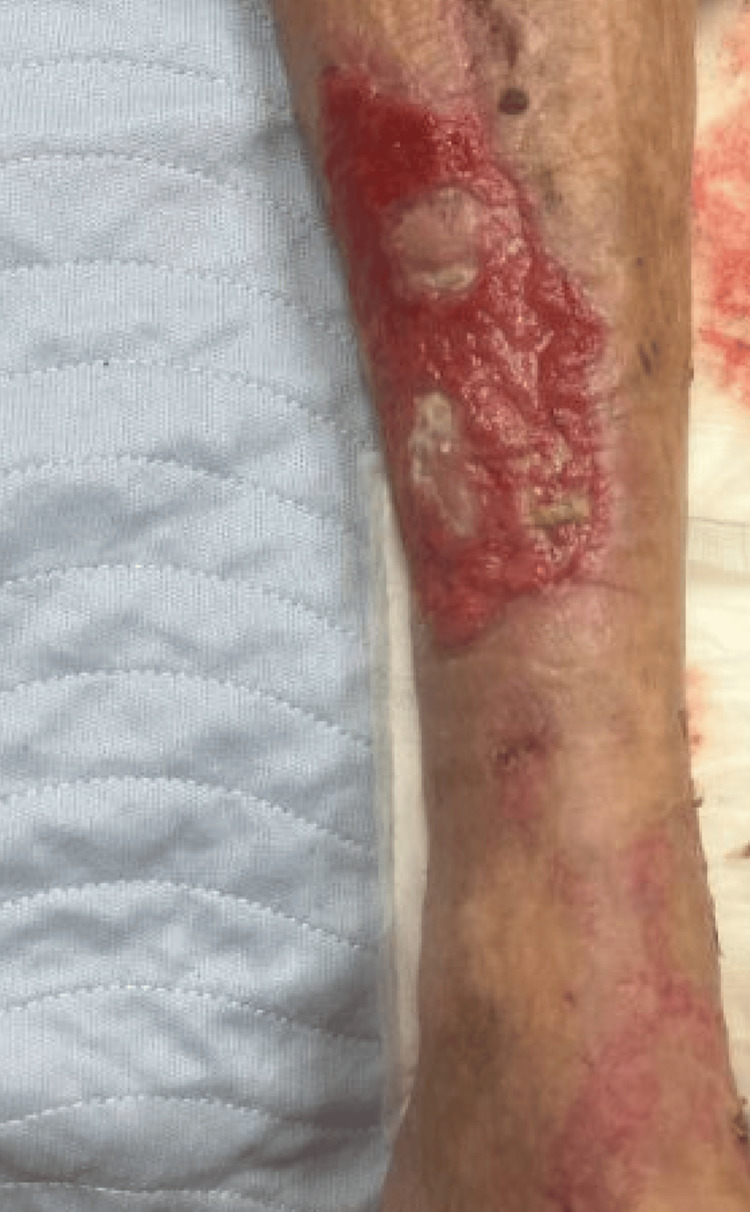
Gross image of the left lower leg showing cutaneous ulcers

A 60-year-old Caucasian male with a past medical history of chronic obstructive pulmonary disease (COPD)/emphysema, congestive heart failure (CHF), gastroesophageal reflux disease (GERD), and hepatitis C virus (HCV) was incidentally diagnosed with CLL in 2016 after new-onset lymphocytosis during HCV antiviral therapy. Initial workup revealed a white blood cell count of 25,000/μL (reference range: 4,000-10,000/μL) with an absolute lymphocyte count of 16,400/μL (reference range: 1,200-4,000/μL), normal red blood cell indices, and a normal platelet count. Peripheral blood smear demonstrated absolute lymphocytosis composed predominantly of small, mature-appearing lymphocytes without significant morphologic atypia. Red cell morphology revealed slight anisocytosis and poikilocytosis with ovalocytes, elliptocytes, occasional burr cells, and mild polychromasia, consistent with borderline normocytic normochromic anemia. Platelet morphology was unremarkable. Flow cytometry of peripheral blood demonstrated a monoclonal B-cell population co-expressing CD19, CD5, CD20, CD22, and FMC7 with restricted kappa light chain expression (kappa/lambda ratio 30.2:1), consistent with a small B-cell lineage malignant lymphoma. Myeloid markers (CD13, CD14, CD33) were negative (<1%). The pharmaceutical team confirmed that the lymphocytosis was not attributable to the antiviral agents. The patient was observed under active surveillance, as lymphocyte counts normalized and the patient was asymptomatic, but clonal B-cell populations persisted on serial flow cytometry, and breakpoint cluster region-Abelson (BCR-ABL) testing was negative. In 2020, the patient developed painful purpura and non-healing ulcers on the lower extremities as seen in Figure 1.

**Figure 2:**
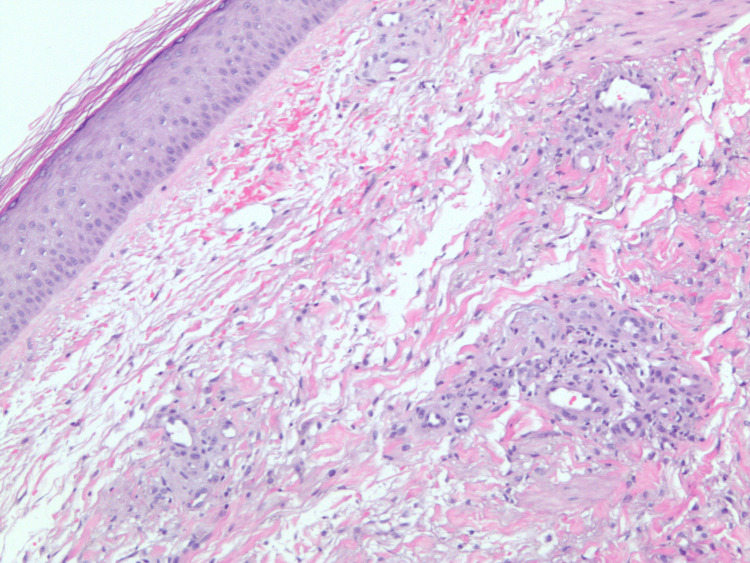
Low-power hematoxylin and eosin (H&E) stain demonstrating leukocytoclastic vasculitis

**Figure 3:**
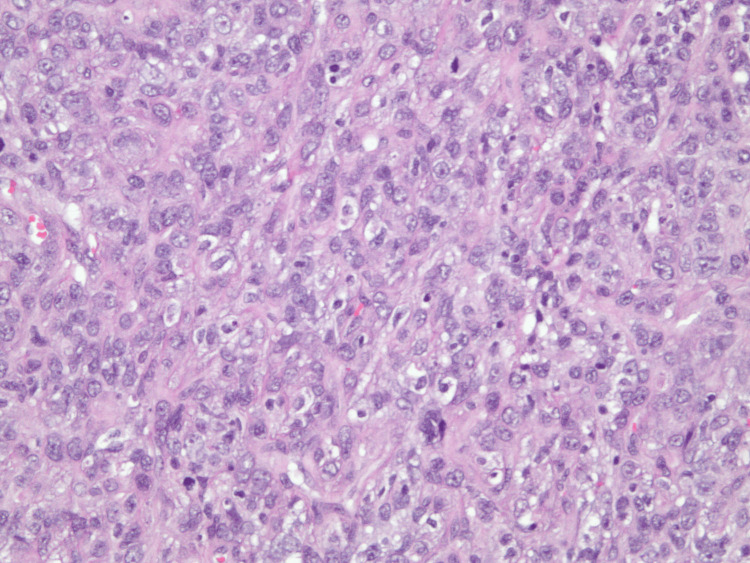
High-power hematoxylin and eosin (H&E) stain showing neutrophil-predominant small-vessel vasculitis

**Figure 4:**
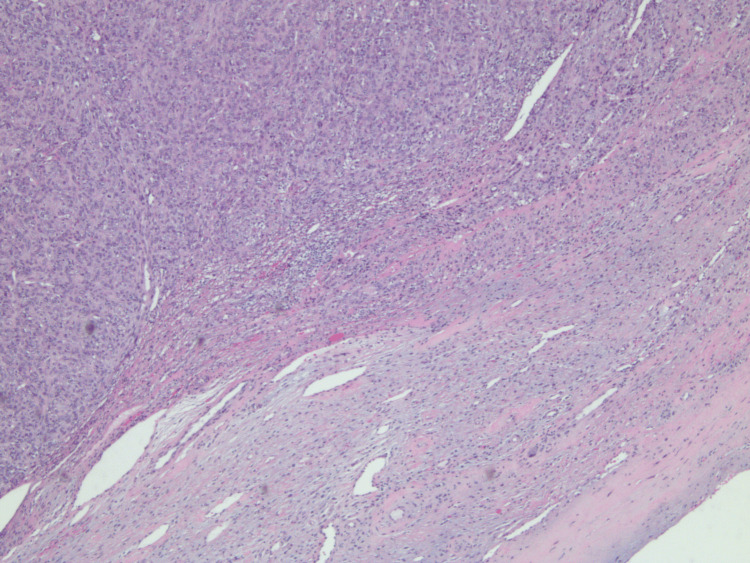
Low-power hematoxylin and eosin (H&E) stain showing perivascular neutrophilic inflammatory infiltrates

C3 was within normal limits; C4 was reduced at <4 mg/dL (reference: 16-38 mg/dL), suggesting complement consumption via the lectin or classical pathway. Skin biopsy confirmed IgA leukocytoclastic vasculitis with perivascular neutrophilic inflammatory infiltrates as seen in Figures 2-4. Direct immunofluorescence (DIF) of the skin lesion revealed strong granular deposition of C3 (4+) and IgA (3+) in superficial dermal capillaries, along with moderate granular IgG deposition (2+). Fibrinogen deposition was observed in superficial dermal capillaries and the perivascular interstitium. The strong IgA and C3 deposition in superficial dermal vessels is diagnostic of IgA vasculitis, while the fibrinogen deposition suggests active vasculitis with ongoing vascular damage. The concurrent IgG deposition, although less prominent than IgA, may reflect the underlying CLL-associated immunoglobulin dysregulation. The patient was started on prednisone, and the disease course was complicated by recurrent infections and steroid toxicities, including hospitalization for pneumonia and development of steroid-induced diabetes. Subsequently, the patient developed varicella-zoster virus (VZV) infection involving the left face and was treated with acyclovir.

A temporary lapse in care and a steroid pause led to readmission of the patient for worsening lower extremity ulcers. Pathology remained consistent for IgA vasculitis, and prednisone was increased from 20 mg to 40 mg daily with a 10 mg taper. Severe ulcer pain was managed with morphine and hydrocodone with varying relief patterns. The patient later developed bilateral lower extremity cellulitis and was treated with linezolid and piperacillin-tazobactam. As lower-extremity ulcers epithelialized, areas of necrotic eschar were noted. The patient was started on Lasix for lower limb edema, prednisone was maintained at 40 mg/day for “probable vasculitis”, and tapering to 20 mg reliably triggered ulcer flares, establishing a clear steroid dependence.

Despite normalized lymphocyte counts, flow cytometry continued to demonstrate clonal B-cell populations, suggesting subclinical CLL activity. The patient remained under multidisciplinary care with hematology, rheumatology, and dermatology. In 2025, the patient was readmitted with acute hypoxemic respiratory failure due to CHF and COPD, superimposed with sepsis due to multifocal methicillin-resistant *Staphylococcus aureus* (MRSA) and extended-spectrum β-lactamase (ESBL) pneumonia, and was treated with ertapenem, Zyvox, and prophylactic Bactrim for *Pneumocystis jirovecii* pneumonia (PJP) due to chronic immunosuppression, high-flow nasal cannula, steroids, and diuresis. Imaging also revealed a 5.7 cm infrarenal abdominal aortic aneurysm with mural thrombus. Despite brief stabilization, the patient had abrupt respiratory decompensation with subsequent epistaxis and a large hemoptysis episode, necessitating rapid response. As the patient was considered high risk for any surgical intervention due to his respiratory status, he was transitioned to comfort care and passed due to respiratory failure.

The patient faced numerous challenges throughout his disease course. CLL’s immunocompromising effects, with hypogammaglobulinemia, predisposed the patient to recurrent serious bacterial infections due to low IgA and IgG subclasses. Prolonged glucocorticoid use resulted in steroid dependence, recurrent infections including pneumonia and cellulitis, impaired wound healing, and eventual respiratory failure. Steroid therapy may paradoxically exacerbate ulcerations in the setting of recurrent infections and poor perfusion.

## Discussion

IgA vasculitis (formerly HSP) is a small-vessel leukocytoclastic vasculitis classically presenting with palpable purpura, arthralgia, renal involvement, and abdominal symptoms, with IgA-dominant immune deposits in the small vessels of the skin and kidneys [1]. This immune complex deposition can be triggered by abnormal IgA production, often in response to antigenic complement stimulation, with the cascade resulting in the “classic clinical tetrad” of palpable purpura, gastrointestinal (GI) manifestations (such as bleeding or abdominal pain), arthralgia, and glomerulonephritis [10].

While classically described in children, adult-onset cases tend to be much more severe and can often indicate an underlying solid/hematologic malignancy that can dysregulate the patient’s immune system, as described in a case series of three patients [11]. Both solid tumors and hematologic malignancies can induce systemic immune dysregulation characterized by persistent immune complex formation, driven by tumor-associated neoantigen exposure and chronic pro-inflammatory signaling (interleukin-6 (IL-6) and tumor necrosis factor-α (TNF-α)). This immune shift promotes IgA production, increases vascular permeability, and impairs clearance of abnormal immunoglobulins, leading to immune-complex deposition and vasculitis [8,12].

In our patient with underlying indolent CLL, IgA vasculitis manifested with painful purpura and non-healing leg ulcers. The defective clonal B-cells in CLL can produce dysfunctional, low-affinity, poorly glycosylated immunoglobulins, including polyclonal and monoclonal IgA, which may fail to undergo proper class switching or tolerance induction [6,13]. Although the clonal B cells in CLL appear morphologically mature, they fail to undergo normal apoptosis and accumulate in the peripheral blood, bone marrow, and lymphoid tissues. This leads to leukocytosis, disruption of normal B-cell maturation, impaired plasma-cell differentiation, and hypogammaglobulinemia, ultimately increasing susceptibility to infections [6,13].

Hematologic malignancies, particularly CLL, predispose to autoimmune complications through a broader range of immune dysregulation, including chronic immune activation, impaired antigen presentation, and loss of normal immune tolerance [14,15]. CLL is therefore associated with significant immune dysregulation, predisposing patients to autoimmune hemolytic anemia, immune thrombocytopenia, and, rarely, pure red cell aplasia, autoimmune neutropenia, and other rare vasculitides as seen in our patient [14,16].

Autoimmune cytopenias (AIC) affect 4-7% of patients with CLL and mainly consist of autoimmune hemolytic anemia and immune thrombocytopenia [16]. Loss of B-cell tolerance causes the production of aberrant, autoreactive immunoglobulins, which activate complement through the alternative and lectin pathways. Impaired clearance of these dysfunctional antibodies nurtures the formation of persistent, circulating type III immune complexes, further driving complement activation and downstream inflammatory injury, leading to leukocytoclastic vasculitis [14]. Diminished complement levels in CLL patients with purpura should prompt suspicion of immune complex-driven vasculitis [9].

Malignant B cells act as dysfunctional antigen-presenting cells, while regulatory T-cell dysfunction allows autoreactive clone expansion. Global B-cell dysregulation reduces normal immunoglobulin production, promotes the persistence of autoreactive B cells, and increases autoantibody production [17]. T-cell exhaustion and pro-inflammatory subset skewing further amplify autoreactivity, and the CLL microenvironment, enriched with IL-10, TGF-β, B-cell activating factor (BAFF), and a proliferation-inducing ligand (APRIL), supports the survival of pathogenic B-cell populations [17]. CLL clones can secrete monoclonal immunoglobulins or light chains mimicking immune complexes, driving complement consumption and vascular inflammation through paraproteinemia. This highlights the diverse effects of persistent B-cell populations on CLL pathology, even with stable lymphocyte counts [17]. The International Prognostic Score for Asymptomatic Early-stage Disease (IPS-E) indicates that only about 8% of low-risk CLL patients require treatment within five years of diagnosis [18]. Current treatment indications include CLL-related complications such as significant anemia or cytopenia, symptomatic lymphadenopathy, hepatosplenomegaly, or refractory autoimmune thrombocytopenia [18].

Unlike many cases where paraneoplastic syndromes correlate with clinical progression, our patient exhibited recurrent vasculitis flares despite an untreated stable CLL. Although lymphocyte counts were stable, flow cytometry demonstrated persistent clonal B-cell populations from 2016 to 2025, suggesting ongoing subclinical CLL. This subclinical CLL-related immune dysregulation might have contributed to vasculitis even in the absence of overt disease, demonstrating that paraneoplastic immune complications can manifest independently and that this would likely be an indication to treat underlying CLL.

Adult IgA vasculitis can be paraneoplastic, and the malignancy is not limited to CLL. Case reports show other types of cancers, such as lung, genitourinary tract, and GI tract cancer, representing the three most solid malignancies, along with non-Hodgkin lymphoma, chronic myelomonocytic leukemia, among others [11,19]. A retrospective study of 14 adult IgA vasculitis cases found that 29% were associated with malignancy, and when combined with 15 additional literature cases, 63% were solid tumors, and 37% were hematologic malignancies [20]. Another study reported that HSP associated with solid-organ malignancies appeared less responsive to standard immunosuppressive therapy unless the underlying cancer was treated simultaneously [11]. For our patient, any attempts to taper prednisone below 20 mg provoked ulcer flares, demonstrating a clear steroid dependence and the difficulties in achieving remission without treating the underlying cause of the vasculitis.

Adult-onset IgA vasculitis presents with nonspecific symptoms and should prompt screening for underlying malignancy. Early recognition enables timely treatment and steroid planning to mitigate infection risk and metabolic toxicity. This case highlights the importance of close monitoring during corticosteroid therapy, coordinated multidisciplinary management, gradual steroid tapering to minimize rebound complications, and early consideration of steroid-sparing therapies in complex or refractory cases. Notably, vasculitis itself may indicate the need for treating the underlying CLL, potentially improving vasculitic outcomes, and continued research is necessary to define better management strategies for this overlap between malignancy and immune-mediated vasculitis.

## Conclusions

This case highlights IgA vasculitis as a rare paraneoplastic manifestation of CLL, presenting with purpura, non-healing ulcers, and systemic inflammatory symptoms. Management is complicated by steroid dependence, infection risk, and impaired wound healing, underscoring the need for early recognition and multidisciplinary care. Adult-onset IgA vasculitis should prompt malignancy screening and consideration of steroid-sparing strategies. Further research is needed to elucidate mechanisms of IgA dysregulation in B-cell malignancies and identify predictive biomarkers.
